# Supplementary material for: Emergent second law for non-equilibrium steady states
Source: Nat Commun. 2022 Aug 29;13:5084. doi: 10.1038/s41467-022-32700-7 (PMC9424242; doi:10.1038/s41467-022-32700-7)
Supplement: Supplementary file 1 — Supplementary Information [file 41467_2022_32700_MOESM1_ESM.pdf]

Supplementary Information for "Emergent second law for non-equilibrium steady states"

Nahuel Freitas[1] and Massimiliano Esposito[1]

[1] *Complex Systems and Statistical Mechanics, Department of Physics and Materials Science, University of Luxembourg, L-1511 Luxembourg, Luxembourg*

PACS numbers:

I. SUPPLEMENTARY NOTE 1

We provide here the details of the example given in the main text, based on the model of a non-equilibrium electronic memory developed in [1, 2]. This is a stochastic model of a low-power CMOS memory cell, whose circuit diagram is shown in Figure 1-(a). This circuit involves two identical CMOS inverters, or NOT gates, connected in a loop. Each inverter is in turn composed of a nMOS transistor and a pMOS transistor. The circuit is subjected to a voltage bias $2V_{\rm dd}$, and it has two degrees of freedom: the voltages v_1 and v_2 at the output of each inverter. The total electrostatic energy of the full circuit is $\Phi(v_1, v_2) = (C/2)(v_1^2 + v_2^2) + CV_{\rm dd}^2$, where C is a value of capacitance characterizing the circuit. The internal electronic entropy $S(v_1, v_2)$ is usually neglected in this kind of circuits, and therefore the entropy production is given by the entropy flow alone: $\Sigma = \Sigma_e = -Q/T$, where $-Q$ is the heat dissipated by the circuit.

Each transistor is modelled as a controlled conduction channel (see details in [1]), and two Poisson rates are associated to conduction events in forward and backward directions. In each conduction event, the voltages $v_{1/2}$ can change by the elementary voltage $v_e = q_e/C$. The Poisson rates can be derived from the I-V curve characterization of the transistors and the local detailed balance conditions [1]. For example, for the pMOS transistor in the first inverter one obtains the following Poisson rates (in subthreshold operation):

$$\begin{aligned} \lambda_+^p(v_1, v_2) &= (I_0/q_e)\, e^{(V_{\rm dd} - v_2 - V_{\rm th})/({\rm n}V_T)} \\ \lambda_-^p(v_1, v_2) &= \lambda_+^p(v_1, v_2)\, e^{-(V_{\rm dd} - v_1)/V_T}\, e^{-(v_e/2)/V_T}, \end{aligned} \tag{1}$$

In the previous equations, $V_T = k_b T/q_e$ is the thermal voltage and I_0, $V_{\rm th}$, and n are parameters characterizing the transistor (respectively known as *specific current*, *threshold voltage*, and *slope factor*). Assuming those parameters are the same for all the transistors, the Poisson rates associated to the nMOS transistor in the first inverter are just

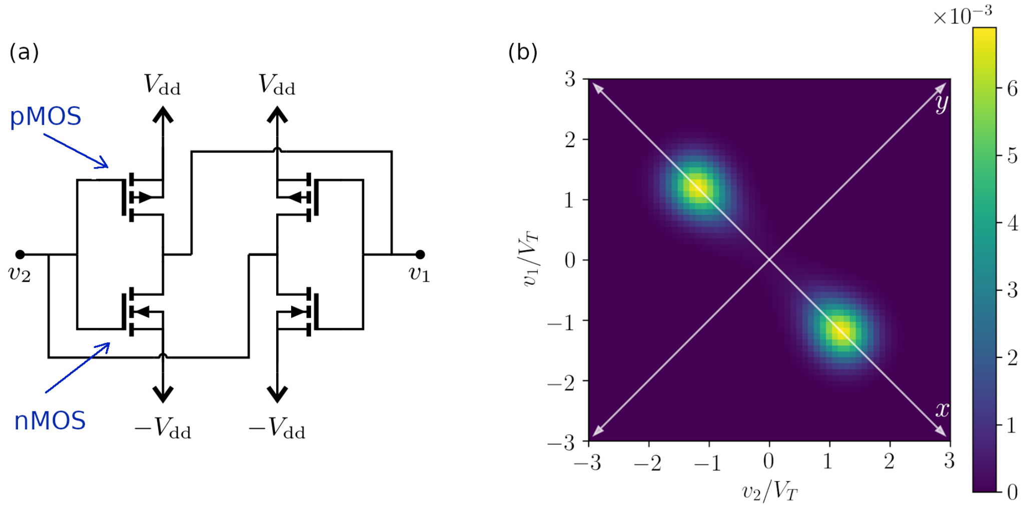

Supplementary Figure 1: (a) CMOS implementation of a bistable logical circuit involving two NOT gates in a loop, where each NOT gate is constructed with one pMOS (top) and one nMOS (bottom) transistor. This is the usual way SRAM memory cells are implemented. (b) 2D histogram of the steady state distribution ($v_e/V_T = 0.1$, $V_{\rm dd}/V_T = 1.3$).

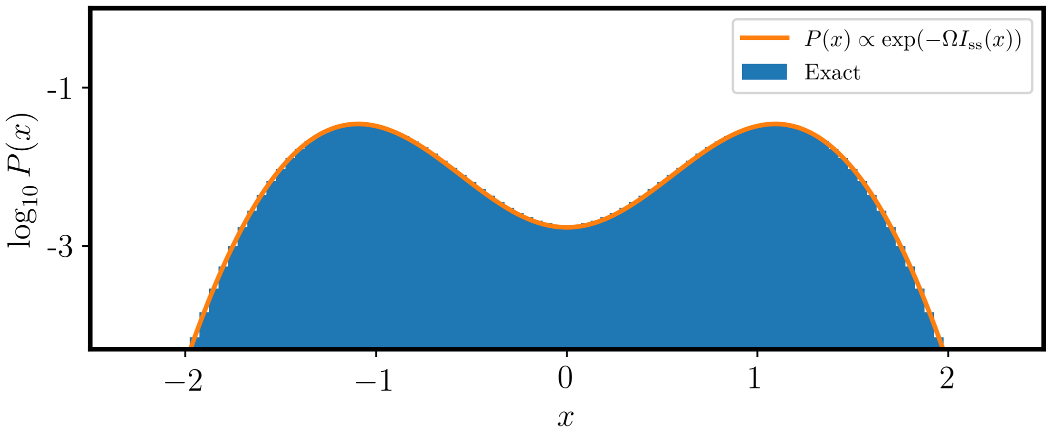

Supplementary Figure 2: Comparison between the exact steady state distribution for x and the LD approximation obtained from the rate function $I_{\rm ss}(x)$ in the main text. ($\Omega = 10$ and $V_{\rm dd} = 1.2 V_T$).

$\lambda^n_\pm(v_1, v_2) = \lambda^p_\pm(-v_1, -v_2)$, while the Poisson rates associated to the transistors in the second inverter are given by $\mu^{n/p}_\pm(v_1, v_2) = \lambda^{n/p}_\pm(v_2, v_1)$. The total rate for a transition $v_1 \to v_1 + v_e$ is $\tilde\lambda_+(v_1, v_2) = \lambda^p_+(v_1, v_2) + \lambda^n_-(v_1, v_2)$, and the total rate for a transition $v_1 \to v_1 - v_e$ is $\tilde\lambda_-(v_1, v_2) = \lambda^p_-(v_1, v_2) + \lambda^n_+(v_1, v_2)$. Then, the state of the system is described by a probability distribution $P(v_1, v_2, t)$ that evolves according to the master equation

$$d_t P(v_1, v_2, t) = P\tilde\lambda_+|_{v_1 - v_e, v_2} + P\tilde\lambda_-|_{v_1 + v_e, v_2} + P\tilde\lambda^*_+|_{v_1, v_2 - v_e} + P\tilde\lambda^*_-|_{v_1, v_2 + v_e} - P(\tilde\lambda_+ + \tilde\lambda_- + \tilde\lambda^*_+ + \tilde\lambda^*_-)|_{v_1, v_2}, \tag{2}$$

where we are using the compact notation $PA|_{v_1, v_2} = P(v_1, v_2, t)\tilde\lambda_\pm(v_1, v_2)$, and $\tilde\lambda^*_\pm(v_1, v_2) = \tilde\lambda_\pm(v_2, v_1)$. One can numerically compute the steady state distribution from the previous master equation by truncating the state space to a finite number of states and computing the eigenvector of zero eigenvalue of the master equation generator, or alternatively by Gillespie simulations of the stochastic dynamics. An example is shown in Figure 1-(b), obtained with the former method.

A. Macroscopic limit

A macroscopic limit can be introduced by considering a particular scaling of the physical dimensions of the transistors. In a MOS transistor there are two characteristic dimensions defined with respect to the conduction channel [3]: the length L and the width W. For fixed L, both the capacitance C and the characteristic current I_0 increase linearly with W. Thus, we can consider $\Omega = V_T/v_e \propto W$ as the adimensional scale parameter in the main text. In the following we consider the adimensional voltages v_1 and v_2, in units of V_T. We also define the scaled rates $\tilde\omega_\pm(v_1, v_2) = \lim_{\Omega \to +\infty} \tilde\lambda_\pm(v_1, v_2)/\Omega$. Then, introducing the large deviations ansatz $P_{\rm ss}(v_1, v_2) \propto \exp(-\Omega I_{\rm ss}(v_1, v_2))$ in the master equation and keeping only the dominant terms in Ω, we obtain the following differential equation for $I_{\rm ss}(v_1, v_2)$:

$$0 = \left(e^{\partial_{v_1} I_{\rm ss}} - 1\right)\tilde\omega_+(v_1, v_2) + \left(e^{-\partial_{v_1} I_{\rm ss}} - 1\right)\tilde\omega_-(v_1, v_2) + \left(e^{\partial_{v_2} I_{\rm ss}} - 1\right)\tilde\omega_+(v_2, v_1) + \left(e^{-\partial_{v_2} I_{\rm ss}} - 1\right)\tilde\omega_-(v_2, v_1). \tag{3}$$

As shown in [2], evaluating the previous equation at $y \equiv v_1 + v_2 = 0$ leads to a differential equation for the reduced rate function $I_{\rm ss}(x)$ associated to the variable $x \equiv v_1 - v_2$ (this is justified by the contraction principle and the fact that the most probable value of y for any value of x is $y = 0$, see Figure 1-(b)). In that way it is possible to derive the expression for $I_{\rm ss}(x)$ given in the main text. In Figure 2, we compare the probability distribution corresponding to that analytical rate function with exact numerical results. The agreement is essentially perfect even if only a few tens of electrons are involved (the scaling parameter is $\Omega = 10$, and $V_{\rm dd} = 1.2 V_T$).

B. Deterministic dynamics

The deterministic equations of motion for the circuit in Figure 1-(a) read:

$$\begin{aligned} CV_T\, d_t v_1 &= \tilde{\lambda}_+(v_1, v_2) - \tilde{\lambda}_-(v_1, v_2) = i(v_1, v_2) - i(-v_1, -v_2) \\ CV_T\, d_t v_2 &= \tilde{\lambda}_+(v_2, v_1) - \tilde{\lambda}_-(v_2, v_1) = i(v_2, v_1) - i(-v_2, -v_1), \end{aligned} \tag{4}$$

where $i(v_1, v_2) = I_0 e^{(V_{\rm dd}/V_T - V_{\rm th}/V_T - v_2)/{\rm n}} \left(1 - e^{-(V_{\rm dd}/V_T - v_1)}\right)$ is the deterministic electric current through the pMOS transistor for given v_1 and v_2. Alternatively, in terms of variables x and y defined above,

$$\begin{aligned} CV_T\, d_t x &= i(x, y) - i(-x, -y) - i(-x, y) + i(x, -y) \\ CV_T\, d_t y &= i(x, y) - i(-x, -y) + i(-x, y) - i(x, -y), \end{aligned} \tag{5}$$

where the change of variables in the function $i(.,.)$ is implicit. Noting that $d_t y|_{y=0} = 0$ for all x, we realize that $y(t) = 0$ for all t if we have $y(0) = 0$. Finally, we note that the total deterministic work rate for given values of votages v_1 and v_2 is $\dot{W} = V_{\rm dd}[i(v_1, v_2) + i(-v_1, -v_2) + i(v_2, v_1) + i(-v_2, -v_1)]$. The scaled work rate defined in the main text is in this case $\dot{w} = \dot{W} v_e / V_T$. The detailed balance drift $\boldsymbol{u}^{(0)}$ and the lowest order work rate $\dot{w}^{(0)}$ are obtained from the previous expressions by evaluating the currents $i(v_1, v_2)$ at $V_{\rm dd} = 0$. Note also that the deterministic dynamics has a natural timescale $\tau = CV_T / (I_0 e^{-V_{\rm th}/{\rm n}V_T})$.

C. Vorticity

For this model, the curl or vorticity defined in the last section of the main text reads:

$$\begin{aligned} f(v_1, v_2) &= d_{v_2} \log(\tilde{\omega}_+(v_1, v_2)/\tilde{\omega}_-(v_1, v_2)) - d_{v_1} \log(\tilde{\omega}_+(v_2, v_1)/\tilde{\omega}_-(v_2, v_1)) \\ &\propto \frac{1}{2} \sinh(V_{\rm dd}) \left(\cosh(v_1 - 2v_2) - \cosh(2v_1 - v_2)\right) \times \\ &\quad {\rm sech}\left(\frac{v_1 - V_{\rm dd}}{2} - v_2\right) {\rm sech}\left(v_1 - \frac{v_2 + V_{\rm dd}}{2}\right) {\rm sech}\left(\frac{v_1 + V_{\rm dd}}{2} - v_2\right) {\rm sech}\left(v_1 - \frac{v_2 - V_{\rm dd}}{2}\right) \end{aligned} \tag{6}$$

II. SUPPLEMENTARY NOTE 2

We consider the coarse-grained jump rates $\tilde{\lambda}(\boldsymbol{n})$ defined in the main text and their macroscopic limit $\tilde{\omega}(\boldsymbol{x}) = \lim_{\Omega\to\infty} \tilde{\lambda}(\Omega \boldsymbol{x})/\Omega$. The deterministic drift is expressed in terms of these rates as

$$\boldsymbol{u}(\boldsymbol{x}) = \sum_\rho \tilde{\omega}_\rho(\boldsymbol{x}) \tilde{\boldsymbol{\Delta}}_\rho = \sum_{\rho>0} (\tilde{\omega}_\rho(\boldsymbol{x}) - \tilde{\omega}_{-\rho}(\boldsymbol{x})) \tilde{\boldsymbol{\Delta}}_\rho, \tag{7}$$

and the macroscopic coarse-grained entropy production rate reads:

$$\dot{\pi}(\boldsymbol{x}_t) \equiv \lim_{\Omega\to\infty} \dot{\Pi}/\Omega = \sum_{\rho>0} (\tilde{\omega}_\rho(\boldsymbol{x}_t) - \tilde{\omega}_{-\rho}(\boldsymbol{x}_t)) \log(\tilde{\omega}_\rho(\boldsymbol{x}_t)/\tilde{\omega}_{-\rho}(\boldsymbol{x}_t)) \tag{8}$$

If the deterministic dynamics emerging in the macroscopic limit has fixed-point attractors, then $\boldsymbol{u}(\boldsymbol{x}^*) = 0$ at the fixed points $\boldsymbol{x}^*$. Whenever the set $\{\tilde{\boldsymbol{\Delta}}_\rho\}$ of distinct jump vectors is linearly independent, $\boldsymbol{u}(\boldsymbol{x}^*) = 0$ implies that $\tilde{i}_\rho(\boldsymbol{x}^*) \equiv \tilde{\omega}_\rho(\boldsymbol{x}^*) - \tilde{\omega}_{-\rho}(\boldsymbol{x}^*) = 0$ for all ρ. As a consequence, the entropy production rate in Eq. (8) vanishes at the fixed points. The linear independence assumption is always satisfied for electronic circuits, but it might fail to hold for some chemical reaction networks.

The fact that the macroscopic coarse-grained entropy production vanishes at the fixed points of the deterministic dynamics does not imply that the model is detailed-balanced at the coarse-grained level. The model will be effectively detailed-balanced if and only if the log-ratio $\tilde{\sigma}_\rho(\boldsymbol{x}) \equiv \log(\tilde{\omega}_\rho(\boldsymbol{x})/\tilde{\omega}_{-\rho}(\boldsymbol{x}))$ can be derived from a gradient, i.e., if $\tilde{\sigma}_\rho(\boldsymbol{x}) = -\tilde{\boldsymbol{\Delta}}_\rho \cdot \nabla \tilde{\phi}(\boldsymbol{x})$ for some function $\tilde{\phi}(\boldsymbol{x})$. Whenever the set $\{\tilde{\boldsymbol{\Delta}}_\rho\}$ is also orthonormal (as it is for the example in the previous section), one can check if $\tilde{\sigma}_\rho(\boldsymbol{x})$ derives from a gradient by checking if the generalized curl $f_{\rho,\rho'}(\boldsymbol{x}) \equiv \partial_{x_\rho} \tilde{\sigma}_{\rho'}(\boldsymbol{x}) - \partial_{x_{\rho'}} \tilde{\sigma}_\rho(\boldsymbol{x})$ vanishes for all ρ, ρ', and $\boldsymbol{x}$.

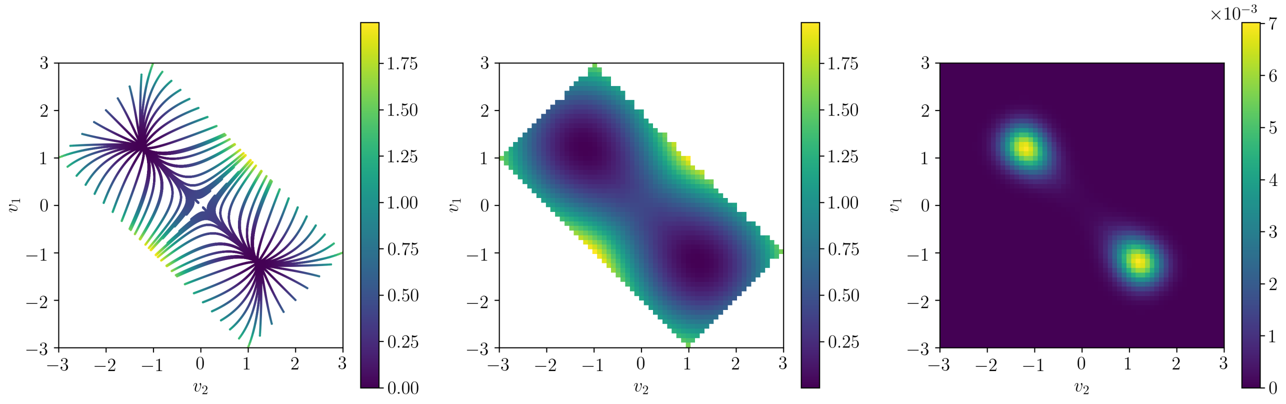

Supplementary Figure 3: (a) A set of deterministic trajectories converging to the fixed points. The color of each point indicates the value $\pi = \int_0^{+\infty} dt'\ \dot{\pi}(\boldsymbol{v}_{t'})$ of the scaled coarse-grained entropy produced during the relaxation from that point until the fixed point, which according to the emergent second law is an upper bound to the true steady state rate function. (b) 2D linear interpolation of the data in (a). (c) Probability distribution obtained as $P_{\rm rec} \propto e^{-I_{\rm rec}/v_e}$, where $I_{\rm rec}$ is the estimation of the rate function in (b). The parameters are the same as in Figure 1-(b).

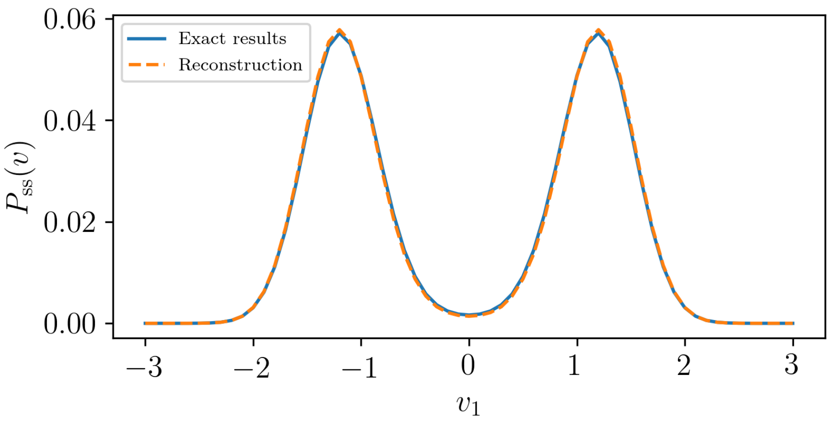

Supplementary Figure 4: Comparison of the partial distribution for the variable v_1 as obtained from the exact steady state of Figure 1-(b) and the reconstructed one in Figure 3-(c).

III. SUPPLEMENTARY NOTE 3

In this section we illustrate how the emergent second law can be employed as an alternative to stochastic simulations (for example the Gillespie algorithm) or spectral methods (finding an eigenvector of the master equation generator) in order to compute non-equilibrium steady state distributions. The strategy is to cover the state space with a sufficiently dense set of deterministic trajectories. Then, a bound for the steady state rate function can be obtained along each of the trajectories. If this bound is obtained from the coarse-grained entropy production, instead of the physical one, we known that at least close to deterministic fixed points the bound will be tight (since by construction the coarse-grained currents vanish at the fixed points, and the bound is tight close to equilibrium). In the particular case of the CMOS memory, we know that the bound is also an accurate approximation of the rate function far away from the fixed point. This procedure is shown in Figure 3. In Figure 3-(a) we show a set of deterministic trajectories, each of which is colored in a way that indicates the value of the bound to the rate function as given by the emergent second law, and that in the following will be considered an estimation of the actual rate function. In Figure 3-(b) we interpolate the data in Figure 3-(a) using the 'griddata' function of the SciPy suite [4] with the 'linear' interpolation method. The rate function reconstructed in this way is employed to produce the probability distribution shown in

Figure 3-(c). The parameters employed are the same as for the exact distribution in Figure 1-(b), and the fidelity between the exact and the reconstructed distribution is very high. As an example, we show in Figure 4 the partial distribution for v_1 according to both results.

IV. SUPPLEMENTARY NOTE 4

The emergent second law presented in the main text can also be understood as a generalized fluctuation-dissipation relation valid arbitrarily out of equilibrium. In this section we make that connection more explicit. The emergent second law states that

$$I_{\rm ss}(\boldsymbol{x}_t) - I_{\rm ss}(\boldsymbol{x}_0) \geq -\int_0^t dt' \dot{\sigma}(\boldsymbol{x}_{t'}) \tag{9}$$

for any deterministic trajectory $\boldsymbol{x}_t$, where $I_{\rm ss}(\boldsymbol{x})$ is the steady state rate function and $\dot{\sigma}$ the scaled macroscopic entropy production rate. The previous inequality is saturated in two cases: to linear order in the non-equilibrium forces, or close to the fixed points if $\dot{\sigma}$ is replaced by the coarse-grained entropy production rate $\dot{\pi}$ (under the conditions discussed in the Supplementary Note 2). In any of those cases, close to a deterministic fixed point $\boldsymbol{x}^*$, we can write:

$$\frac{1}{2}\boldsymbol{x}_0^T \cdot C \cdot \boldsymbol{x}_0 + \mathcal{O}(|\boldsymbol{x}_0|^3) = \int_0^{+\infty} dt' \dot{\sigma}(\boldsymbol{x}_{t'}) \tag{10}$$

where $C_{i,j} = \partial_{x_i,x_j} I_{\rm ss}(\boldsymbol{x}^*)$. To obtain the previous equation from Eq. (9), we consider trajectories that start at $\boldsymbol{x}_0$ and end at the fixed point $\boldsymbol{x}^*$ after infinite time, and expand the rate function $I_{\rm ss}(\boldsymbol{x}_0)$ to second order around the fixed point, noting that both the rate function and its gradient vanish at the fixed point. Note that C is the scaled covariance matrix of the Gaussian fluctuations around the fixed point, so Eq. (2) is already a fluctuation-dissipation relation.

The deterministic dynamics is $d_t\boldsymbol{x}_t = \boldsymbol{\mu}(\boldsymbol{x}_t)$, which close to the fixed point can be approximated as $d_t\boldsymbol{x}_t = -B \cdot \boldsymbol{x}_t$, where the matrix B has components $B_{i,j} = -\partial_{x_j}\mu_i(\boldsymbol{x}^*)$ (if the fixed point is stable then B is positive definite). The dynamics can then be approximated as $\boldsymbol{x}_t = e^{-Bt}\boldsymbol{x}_0$. In both cases mentioned above the entropy production rate is exactly zero at the fixed point, and that is also its minimum value, so its gradient also vanishes. Then, $\dot{\sigma}(\boldsymbol{x}_t) \simeq \boldsymbol{x}_t^T \cdot S \cdot \boldsymbol{x}_t/2$, where $S_{i,j} = \partial_{x_i,x_j}\dot{\sigma}(\boldsymbol{x}^*)$. Puting everything together we arrive at:

$$C = \int_0^{+\infty} e^{-B^T t} S e^{-Bt} \tag{11}$$

which gives us the typical fluctuations around the fixed point in terms of the dynamics and the dissipation around it. We finally note that the previous equation is the formal solution to the Lyapunov equation

$$0 = B^T C + CB + S. \tag{12}$$

Supplementary References

[1] Nahuel Freitas, Jean-Charles Delvenne, and Massimiliano Esposito. Stochastic Thermodynamics of Nonlinear Electronic Circuits: A Realistic Framework for Computing Around kT. *Phys. Rev. X*, 11(3):031064, Sep 2021.

[2] Nahuel Freitas, Gianmaria Falasco, and Massimiliano Esposito. Linear response in large deviations theory: a method to compute non-equilibrium distributions. *New Journal of Physics*, 23(9):093003, Sep 2021.

[3] Yannis Tsividis and Colin McAndrew. *Operation and Modeling of the MOS Transistor*. Oxford Univ. Press, 2011.

[4] Pauli Virtanen, Ralf Gommers, Travis E. Oliphant, Matt Haberland, Tyler Reddy, David Cournapeau, Evgeni Burovski, Pearu Peterson, Warren Weckesser, Jonathan Bright, Stéfan J. van der Walt, Matthew Brett, Joshua Wilson, K. Jarrod Millman, Nikolay Mayorov, Andrew R. J. Nelson, Eric Jones, Robert Kern, Eric Larson, C J Carey, İlhan Polat, Yu Feng, Eric W. Moore, Jake VanderPlas, Denis Laxalde, Josef Perktold, Robert Cimrman, Ian Henriksen, E. A. Quintero, Charles R. Harris, Anne M. Archibald, Antônio H. Ribeiro, Fabian Pedregosa, Paul van Mulbregt, and SciPy 1.0 Contributors. SciPy 1.0: Fundamental Algorithms for Scientific Computing in Python. *Nature Methods*, 17:261–272, 2020.
